# Nitrate supplementation of rations based on rice straw but not Pangola hay, improves growth performance in meat goats

**DOI:** 10.5713/ajas.20.0254

**Published:** 2020-08-24

**Authors:** Siwaporn Paengkoum, Jiravan Khotsakdee, Pramote Paengkoum, Thomas Schonewille, Chalermpon Yuangklang

**Affiliations:** 1 Faculty of Science and Technology, Nakhon Ratchasima Rajabhat University, Nakhon Ratchasima 30000, Thailand; 2 Faculty of Sciences and Liberal Arts, Rajamangala University of Technology Isan, Nakhon Ratchasima 30000, Thailand; 3 School of Animal Technology and Innovation, Suranaree University of Technology, Nakhon Ratchasima, 30000, Thailand; 4 Department of Farm Animal Health, Faculty of Veterinary Medicine, Utrecht University, Utrecht 3584, The Netherlands

**Keywords:** Nitrate, Rice Straw, Pangola Hay, Digestibility, Goats

## Abstract

**Objective:**

Supplemental nitrate is known to be an effective tool to mitigate methane emission by ruminants. Based on theoretical considerations, supplemental nitrate can improve but also deteriorate the growth performance. The overall effect of supplemental nitrate on growth performance, however, is not yet known. The objective of the current study was therefore to evaluate the effect of a higher dose of NO_3_^−^ on overall growth performance when feeding either Pangola grass hay or rice straw.

**Methods:**

Thirty-two crossbred, 3-month-old Thai native×Anglo-Nubian crossbred male goats were used. The experiment had a 2×2 factorial design with an experimental period of 60 days. Eight goats were randomly allocated to each dietary treatment, i.e. a ration containing either Pangola hay (*Digitaria eriantha Steud*) or rice straw (*Oryza Sativa*) as a source of roughage, supplemented with a concentrate containing either 3.2% or 4.8% potassium nitrate. The rations were formulated to be isonitrogenous. The animals were weighed at the start of the experiment and at days 30 and 60. Feces were collected during the last five days of each 30-day period.

**Results:**

High-nitrate increased overall DM intake by approximately 3%, irrespective the source of roughage, but only the goats fed a rice straw-based ration responded with an increase in body weight (BW). Thus, the overall feed conversion ratio (kg feed/kg BW gain) was influenced by roughage source ×nitrate and decreased by almost 60% when the goats were fed rice straw in combination with a high versus a low dietary nitrate content. The digestibility of macronutrients was only affected by the source of roughage and the digestibility of organic matter, crude protein, and neutral detergent fibre was greater when the goats were fed Pangola hay.

**Conclusion:**

It was concluded that the replacement of soybean meal by nitrate improves the growth performance of meat goats fed rations based on rice straw, but not Pangola hay.

## INTRODUCTION

Enteric methane (CH_4_) is a potent greenhouse gas and the emission that originates from ruminant production is considered relevant [1]. The vast majority of CH_4_ production by ruminants is caused by the anaerobic fermentation of organic matter (OM) in the rumen [2] because the synthesis of volatile fatty acids is inevitably associated with that of hydrogen and therefore of CH_4_ [3].

Nitrate (NO _3_^−^) has been shown to effectively suppress CH_4_ methane production [4–6] because NO_3_^−^ can be reduced to ammonia (NH_3_) and therefore acts as a hydrogen sink [7]. Thus, in rations with limiting crude protein (CP) contents, supplemental NO_3_^−^ can be considered as an alternative source of nitrogen for microbial protein synthesis [8,9]. Supplemental NO_3_^−^ can therefore potentially enhance animal production in CP limiting rations and at same time reduce methane emission. On the other hand, the process of NO_3_^−^ reduction competes with ruminal propionogenesis [10] because propionate also, can act as a hydrogen sink [3]. There are indications that a NO_3_^−^ induced redirection of energy to NH_3_ [11] instead of propionate, would not favor animal production [12] especially in the case where poor quality rations are fed. From this perspective, NO_3_^−^ may not be suitable to mitigate methane emission because tropical roughages typically are of a poor quality [13]. The overall net effect of supplemental NO_3_^−^ on animal production, however, is difficult to predict.

To the best of the authors’ knowledge however, there are currently no studies reporting on the effect of extra NO_3_^−^ on the growth performance in meat goats. In Thailand, Pangola grass (hay) is the preferred type of roughage in goat nutrition because it is generally perceived as a high-quality roughage when compared to rice straw. The latter is typically fed during periods of feed scarcity. The objective of the current study was therefore to evaluate the effect of a higher dose of NO_3_^−^ on overall growth performance when feeding either Pangola grass hay or rice straw.

## MATERIALS AND METHODS

### Ethical considerations

The current experiment was approved by the Animal Ethics Committee of Suranaree University of Technology Isan (approval number, RU3-303-55-01) and based on the Ethics of Animal Experimentation of the National Research Council of Thailand.

### Animals, experimental design and housing

Thirty-two crossbred, 3-month-old Thai native×Anglo-Nubian crossbred male goats were used. The experiment had a 2×2 factorial design with an experimental period of 60 days. Eight goats were randomly allocated to each dietary treatment, i.e. a ration containing either Pangola hay (*Digitaria eriantha Steud*) or rice straw (*Oryza sativa*) as a source of roughage, supplemented with a concentrate containing either 3.2% or 4.8% potassium nitrate (Table 1). The experiment was preceded by a 14-day pre-experimental period in which the animals were fed a ration based on Pangola hay. Throughout the experiment, the goats were housed in individual pens with slatted floors and the animals had free access to fresh water and a mineral block.

### Experimental rations

The roughages and concentrates were offered at 3.5% and 1.5% of body weight (BW), respectively, irrespective either source. The feedstuffs were offered daily in two equal portions at 08:00 and 16:00 h. Feed refusals, if present, were collected and weighed daily before the morning feeding so as to determine the actual daily feed intake. The animals were weighed at the start of the experiment and at days 30 and 60 so as to monitor BW and to adjust the amounts of feed offered. Within the two 30-day periods, each individual animal was therefore offered a fixed amount of dry matter (DM).

Pangola grass was harvested after 45 days of regrowth and sun dried for 5 days. After drying, the moisture content was found to be 125 g/kg and the DM had the following chemical composition (g/kg): crude ash, 84; CP, 73; ether extract (EE), 19; neutral detergent fiber (NDF), 734; acid detergent fiber (ADF), 359. The rice straw, with a moisture content of 78 g/kg, was chemically composited as follows (g/kg DM): crude ash, 123; CP, 33; EE, 10; NDF, 734; ADF, 523. The ingredient and chemical composition of the experimental rations is shown in Table 2. The values on the chemical composition in Table 2 are based on the actual mean DM intakes of the experimental feedstuffs as calculated over the 60 days.

### Collection of samples

The experimental feedstuffs were sampled on the last five days of each 30-day period of the experiment. Then, the feedstuff samples were dried at 70°C for 72 hours, ground to pass a 1 mm sieve, pooled across the 5 sampling days and subsequently stored in sealed plastic bags at ambient temperature (25°C) until analysis. Feces also were collected during the last five days of each 30-day period. The feces from individual goats were quantitatively collected with the use of a plastic sheet which was spread below the slatted floors. The daily feces production of each goat was stored at −18°C. At the end of the 5 days collection period, the stored daily feces collections were thawed, mixed thoroughly and sampled. The samples were dried at 70°C for 72 hours, ground to pass a 1 mm sieve and stored in sealed plastic bags at ambient temperature (25°C) until analysis.

### Chemical analysis

The ash content of the experimental feedstuffs was analyzed by combustion at 600°C for 6 hours. Nitrogen contents were determined by the macro Kjeldahl method [14]; a factor of 6.25 was used to convert nitrogen into CP. The EEs of the feedstuffs were prepared according to the AOAC [14]; the solvent was evaporated, and the residue was weighed. The NDF and ADF contents of the rations were analyzed according to the method of [15] and the values are expressed without residual ash.

### Statistical analysis

All data were subjected to analysis of variance using the general linear model procedure in SAS [16], using the model:

$${\text{Y}}_{\text{ij}}=\mu +{\text{RS}}_{\text{i}}+{\text{N}}_{\text{j}}+{\left(\text{RS}\times \text{N}\right)}_{\text{ij}}+{\text{e}}_{\text{ij}}$$

where Y_ij_ = a response variable (e.g. growth performance, nutrient digestibility); μ = overall mean; RS_i_ = roughage source (*i* = Pangola hay or rice straw); F_j_ = level of dietary nitrate (*j* = low or high); (RS×N)_ij_ = interaction term between RS and level of nitrate; and e_ij_ = residual error. Both, the RS and level of nitrate (N) were set as fixed factors in the statistical model. Throughout, the level of statistical significance was pre-set at p≤0.05.

## RESULTS

### Growth performance

During the first 30-day period, DM-intake was similar between treatments (Table 3) but the goats fed Pangola hay versus rice straw had a significantly (p<0.05) greater gain in BW (Table 3). Next to the source of roughage, BW was also affected by the nitrate content of the ration (p<0.05) but the effect of a high dietary nitrate content was only observed in the goats fed rice straw (p<0.05). The growth rate of the goats was likewise affected by the dietary treatments (p<0.05). The lowest values (p<0.05) on the feed conversion ratio (FCR, g feed/g gain) were found when the animals were fed Pangola hay but the FCR was not affected by the high dietary nitrate content. In contrast, the FCR of the goats fed rice straw was found to be ~3 times lower when the ration contained the high nitrate content.

During the second half of the experiment (i.e. from day 31 to day 60) the DM intake (Table 3) was affected by an interaction between RS and the nitrate content of the ration (p≥0.05). The greatest DM intakes (p<0.05) were observed when the goats were fed Pangola hay and the ration contained a greater amount of nitrate. The rice straw fed goats had a ~11% lower BW at the start of the second half of the experiment and showed a lower gain in BW and growth rate compared to the goats fed Pangola hay (p<0.05). Compared to the first 30 days of the experiment, BW gain and growth rate were likewise affected by nitrate and the interaction between RS and the dietary nitrate content but the differences were only borderline statistically significant.

Overall, from day 0 to day 60, the DM intake was only af fected by both RS and the dietary nitrate content while the gain in BW, final BW and the growth rate were significantly affected by the interaction between RS and the dietary nitrate content. The overall FCR also was influenced by RS× nitrate and decreased by almost 60% when the goats were fed rice straw in combination with a high versus a low dietary nitrate content.

### Macronutrient digestibility

Throughout the experiment, the digestibility of macronutrients (Table 4) was not affected by the interaction between RS and nitrate (p<0.05). The digestibility of NDF tended (p = 0.073) to be affected by a high dietary nitrate content only in the second half of the experiment. The digestibility of ADF was likewise affected and across the two experimental periods, a high dietary nitrate content increased the ADF digestibility by almost 5 percentage units (p<0.05). The source of roughage significantly affected the digestibility’s of OM, CP, and NDF and all values were found to be greater (p<0.05) when the goats were fed Pangola hay. In contrast, the digestibility of ADF was greater (p<0.05) when the goats were fed rice straw instead of Pangola hay.

## DISCUSSION

To the best of the our knowledge the current results are the first showing that a high versus a low dietary nitrate content improves the growth performance of meat goats fed rice straw. Interestingly, the high-nitrate induced improvement of FCR was not observed when the goats were fed Pangola hay (Table 3). Although FCR was systematically improved in goats fed the high-nitrate, rice straw-based rations, during the second half of the experiment the difference in FCR failed to reach statistical significance. The data however, are clearly in line with the FCR-values obtained from day 1 to 30 but the variation was aberrantly much greater when compared with the variation in FCR values during the first half of the experiment. It is therefore most likely that the probability of type-1 error was unfavourably affected by the high variation in FCR during the second half of the experiment.

The observed interaction between RS and high dietary ni trate is not easy to explain but the data are in line with the idea that nitrate enhances the growth of rumen bacteria and therefore renders a greater amount of microbial protein due to the reduction of NO_3_^−^ to NH_3_ in the rumen environment [17]. Although rations were formulated to be iso-nitrogenous within RS, the amount of N available for microbial growth was most likely not similar between the nitrate treatments because ~15% of the protein in soybean meal is not degraded in the rumen environment [18] while virtually all nitrate is reduced to NH_3_ [19]. It is well established that there is a sharp increase in the growth rate of rumen microbes when the amount of available N becomes greater than 50 mg/L [20]. Thus, in the case of the rice straw-based rations the latter might have been highly relevant. The current reasoning implies that in the Panola based rations, the issue of N supply to rumen bacteria was not relevant. Finally, the current data do not support the idea that nitrate negatively affects the growth performance with a nitrate induced reduction of rumen propionate formation [21,22]. Needless to mention that the afore mentioned notions concerning the underlying mechanism on the observed nitrate induced improvement of FCR are speculative and that further studies are required for confirmation.

In the current experiment, neither DM-intake nor nutri ent digestibility was influenced by RS×N. Thus, it is difficult to see that the improved growth performance of the meat goats fed rice straw can be explained by DM intake or the total tract digestibility of nutrients. However, high-nitrate as such increased overall DM intake when the goats were fed rice straw, but quantitatively the effect was rather small when compared with low-nitrate (i.e. ~3%). It therefore appears that, with the exception of N, high versus low nitrate did not affect the amounts of nutrients available to support maintenance and growth of the meat goats. The current trial does not provide clues to explain the stimulatory effect of nitrate on DM intake but [5] has reported that nitrate can cause a reduction in heat production. Since the current study was conducted under tropical conditions, it can be speculated that a reduced heat production explains the somewhat greater DM intake when high nitrate was fed.

With the exception of ADF, apparent nutrient digestibility was found to be greater when the goats were fed Pangola hay instead of rice straw. Clearly, these results are in line with expectation because it is widely accepted that the digestibility of Pangola hay is superior to that of rice straw [23]. However, the ADF digestibility was found to be greater when the goats were fed rice straw. Surprisingly, NDF digestibility remained unchanged when high nitrate was fed. It can therefore be speculated that under high nitrate conditions, the fermentation of cellulose is enhanced. Perhaps, ingestion of the high-nitrate rations affected the rumen ecosystem [19] and as such induced a greater fermentation of cellulose. Unfortunately, the current paper does not provide clues to substantiate the latter suggestion.

The current rations contained either 0.8 (low nitrate) or 1.2% of DM (high nitrate) and such values can be lethal in unadapted ruminants [24], but adaptation apparently enabled rumen bacteria to increase in numbers or increase their nitrite-reducing capacity. In the current study however, the animals were slowly adapted to increased levels of nitrate, 3.2% or 4.8% which most likely enabled the rumen bacteria to sufficiently increase their capacity to reduce nitrite [5]. Moreover, in the case the current nitrate load is expressed as g/kg^0.75^/d, values are calculated to be 0.6 and 0.9 g NO_3_^−^/kg^0.75^/d for the low- and high nitrate rations, respectively. A considerably greater dosage (i.e. 1.6 g NO_3_^−^/kg^0.75^/d) in their methane mitigating rations and they reported slightly elevated levels of methemoglobin [5]. It therefore appears that the nitrate levels used in the current study can be considered non-toxic.

## CONCLUSION

The replacement of soybean meal by nitrate improved the growth performance of meat goats fed rations based on rice straw, but not Pangola hay.

## Figures and Tables

**Table 1:** The ingredient and chemical composition of the experimental concentrates

Items	Low nitrate	High nitrate
Ingredient composition (% as fed)		
Dry cassava distiller’s meal	32.0	35.0
Dry corn distiller’s grains	15.0	12.0
Rice bran	10.0	10.0
Wheat bran	10.0	10.0
Molasses	7.0	7.0
Sunflower oil	6.0	6.0
Soybean meal	14.5	12.9
Potassium nitrate	3.2	4.8
Premix1)	2.3	2.3
Analyzed composition		
Dry matter (g/kg)	922.0	921.5
Crude ash (g/kg DM)	71.2	71.4
Crude protein (g/kg DM)	153.4	153.4
Ether extract (g/kg DM)	62.5	61.5
Neutral detergent fiber (g/kg DM)	425.0	405.0
Acid detergent fiber (g/kg DM)	263.0	256.5
Non-structural carbohydrates (g/kg DM)2)	288.0	308.4

DM, dry matter.

1) The premix consisted of (unit/kg): Ca, 8.5 g; P, 6 g; K, 9.5 g; Mg, 2.4 g; Na, 2.1 g; Cl, 3.4 g; S, 3.0 g; Co, 0.16 mg; Cu, 100 mg; I, 1.3 mg; Mn, 64 mg; Zn, 64 mg; Fe, 64 mg; Se, 0.45 mg; vitamin A, 5,000 IU; vitamin D_3_, 2,200 IU; vitamin E, 15 IU.

2) Calculated as: 1,000-neutral detergent fiber-crude protein-ether extract-crude ash.

**Table 2:** The ingredient and chemical composition of the experimental rations1)

Items	Pangola hay		Rice straw	
	
	Low nitrate	High nitrate	Low nitrate	High nitrate
Ingredient composition (g DM/d)				
Pangola hay2)	416	439	-	-
Rice straw3)	-	-	394	410
Concentrate, low nitrate	253	-	242	-
Concentrate, high nitrate	-	252	-	245
Total intake	669	692	637	656
Analyzed composition (g/kg DM)				
OM	920.8	920.6	896.7	896.3
CP	103.4	102.4	78.8	78.2
EE	35.4	34.5	30.0	29.3
NDF	617.2	614.0	634.4	629.0
ADF	322.7	321.6	424.1	423.3
NSC4)	164.8	169.7	153.6	159.8
Nitrate	8.0	11.6	8.1	11.9

DM, dry matter; OM, organic matter; CP, crude protein; EE, ether extract; NDF, neutral detergent fiber; ADF, acid detergent fiber; NSC, non-structural carbohydrates.

1) The data are on based the actual mean DM intakes of the experimental feedstuffs as calculated over 60 days.

2) The chemical composition of Pangola grass hay with a moisture content of 125 g/kg was as follows (g/kg DM): crude ash, 84; CP, 73; EE, 19; NDF, 734; ADF, 359; NSC, 90.

3) The chemical composition of rice straw with a moisture content of 78 g/kg was as follows (g/kg DM): crude ash, 123; CP, 33; EE, 10; NDF, 734; ADF, 523; NSC, 71.

4) Calculated as: 1,000–neutral detergent fiber–crude protein–ether extract–crude ash.

**Table 3:** Feed intake and growth performance in goats after the feeding of the experimental rations for 60 days

Items	Roughage source (RS) Level of nitrate (N)	Pangola hay		Rice straw		SEM	p-value		
		
		Low	High	Low	High		N	RS×N	
Day 1 to 30									
Mean DM intake (g/d)		623	622	615	620	8.0	0.526	0.847	0.708
Body weight (kg)									
Initial		17.4^a^	17.6^a^	16.8^ab^	16.4^b^	0.22	<0.001	0.609	0.204
Gain		2.3^a^	2.0^a^	0.5^c^	1.3^b^	0.08	<0.001	<0.001	<0.001
Growth rate (g/d)		75.0^a^	67.9^a^	15.6^b^	43.3^c^	2.60	<0.001	<0.001	<0.001
FCR1) (kg/kg)		8.4^b^	9.3^b^	45.0^a^	14.8^b^	2.84	<0.001	<0.001	<0.001
Day 31 to 60									
Mean DM intake (g/d)		717^b^	761^a^	658^c^	691^bc^	11.3	<0.001	0.002	0.618
Body weight (kg)									
Day 31		19.7^a^	19.6^a^	17.3^b^	17.7^b^	0.22	<0.001	0.391	0.307
Gain		2.2^a^	2.2^a^	1.0^c^	1.4^b^	0.11	<0.001	0.050	0.063
Growth rate (g/d)		73.8^a^	74.2^a^	31.7^c^	46.7^b^	3.77	<0.001	0.050	0.063
FCR (kg/kg)		9.8	10.4	48.5	14.9	11.26	0.070	0.162	0.148
Day 1 to 60									
Mean DM intake (g/d)		669^ab^	692^a^	637^c^	656^bc^	8.0	<0.001	0.015	0.824
Body weight (kg)									
Gain		4.5^a^	4.3^a^	1.4^c^	2.7^b^	0.15	<0.001	0.001	<0.001
Final2)		21.9^a^	21.9^a^	18.2^c^	19.1^b^	0.23	<0.001	0.001	<0.001
Growth rate (g/d)		74.4^a^	71.0^a^	23.6^c^	45.0^b^	2.47	<0.001	0.001	<0.001
FCR (kg/kg)		7.9^b^	9.8^b^	36.4^a^	14.7^b^	4.67	0.001	0.043	0.018

SEM, standard error of the mean; DM, dry matter; FCR, feed conversion ratio.

1) Calculated as kg DM feed/kg body weight gain.

2) Statistically analyzed using initial body weight as a covariate.

a–c Different superscripts indicate statistical difference within each row (p<0.05).

**Table 4:** Digestibility (% of intake) of organic matter (OM), neutral detergent fibre (NDF), acid detergent fibre (ADF), and crude protein (CP) in goats after the feeding of the experimental rations for 60 days

Items	Roughage source (RS) Level of nitrate (N)	Pangola hay		Rice straw		SEM	p-value		
		
		Low	High	Low	High		RS	N	RS×N
Day 25 to 30									
OM		73.9^a^	74.2^a^	68.4^b^	70.1^b^	1.19	<0.001	0.405	0.557
NDF		62.8^ab^	64.7^a^	58.6^b^	60.8^ab^	1.51	0.012	0.191	0.922
ADF		49.5^c^	51.8^b^	56.2^ab^	59.4^a^	1.72	<0.001	0.122	0.805
CP		64.1^a^	63.2^a^	52.9^b^	51.4^b^	1.99	<0.001	0.548	0.879
Day 55 to 60									
OM		77.5^a^	76.8^a^	72.2^b^	75.0^ab^	1.00	0.002	0.319	0.089
NDF		67.8^a^	69.2^a^	62.9^b^	66.0^b^	1.20	0.003	0.073	0.503
ADF		56.3^b^	58.0^b^	60.8^ab^	64.4^a^	1.44	0.001	0.088	0.508
CP		70.2^a^	68.4^b^	57.6^b^	59.5^b^	1.89	<0.001	0.974	0.350
Overall means									
OM		75.5^a^	75.5^a^	70.3^b^	72.5^ab^	0.97	<0.001	0.263	0.275
NDF		65.1^a^	67.0^a^	60.8^b^	63.4^ab^	1.20	0.003	0.073	0.773
ADF		52.5^c^	54.9^bc^	58.5^ab^	61.9^a^	1.35	<0.001	0.046	0.719
CP		66.9^a^	65.8^a^	55.3^b^	55.4^b^	1.84	<0.001	0.816	0.745

SEM, standard error of the mean; OM, organic matter; NDF, neutral detergent fibre; ADF, acid detergent fibre; CP, crude protein.

a–c Different superscripts indicate statistical difference within each row (p<0.05).
